# The educational gradient in dental caries experience in Northern- Norway: a cross-sectional study from the seventh survey of the Tromsø study

**DOI:** 10.1186/s12903-023-03487-w

**Published:** 2023-10-24

**Authors:** Silje Navjord Moltubakk, Birgitta Jönsson, Marko Lukic, Lina Stangvaltaite-Mouhat

**Affiliations:** 1 Department of Clinical Dentistry, Faculty of Health Sciences, UiT The Arctic University of Norway, Tromsø, Norway; 2 The Public Dental Health Service Competence Centre of Northern Norway, Tromsø, Norway; 3 Department of Periodontology, Institute of Odontology, The Sahlgrenska Academy, University of Gothenburg, Gothenburg, Sweden; 4 Department of Community Medicine, Faculty of Health Sciences, UiT The Arctic University of Norway, Tromsø, Norway; 5 Oral Health Centre of Expertise in Eastern Norway, Oslo, Norway

**Keywords:** Oral health, Dental caries, Educational gradient, Social inequality

## Abstract

**Background:**

Although, studies from Norway indicate a reduction in dental caries experience, in Northern-Norway this non-communicable oral condition is still prevalent. There is conflicting evidence of presence of social inequalities in dental caries in an adult population. Therefore, the aim of this study was to assess an association between educational level and dental caries experience in adults in urban Tromsø municipality, Northern-Norway, using The World Health Organization (WHO) Commission on Social Determinants of Health (CSDH) framework of health determinants.

**Methods:**

Data from 3752 participants having recorded dental caries status and educational level in the seventh survey of the Tromsø Study: Tromsø7 were included. Dental status was examined clinically as decayed-, missing-, filled-teeth (DMFT score). For statistical analyses DMFT score was grouped into lower (DMFT < 19) and higher (DMFT ≥ 20). Educational level was obtained from a questionnaire and categorized as primary/partly secondary education, upper secondary education, tertiary education, short and tertiary education, long. Data on social and intermediary determinants was also self-reported. Univariable and multivariable binary logistic regression analyses were applied.

**Result:**

This study included 1939 (52%) women and the mean age of the participants was 57.11. The mean DMFT score was 18.03. The odds of having higher DMFT score followed a gradient based on educational level. Participants who reported lower than secondary education had 2.06 -fold increased odds of having higher DMFT score than those with tertiary education, long (OR: 2.06, 95% CI: 1.50–2.83). Those with upper secondary education had 60% higher odds of having higher DMFT score (OR: 1.60, 95% CI: 1.21–2.11), and those with tertiary education, short had 66% higher odds of having higher DMFT score (OR: 1.66, 95% CI: 1.24–2.22).

**Conclusion:**

The current cross-sectional study suggested an educational gradient in dental caries experience in an adult population of Northern- Norway. Further studies validating our results and investigating mechanisms of educational inequalities in oral health are warranted.

**Supplementary Information:**

The online version contains supplementary material available at 10.1186/s12903-023-03487-w.

## Background

Dental caries is a non-communicable oral health condition and considered a major public health problem [1]. Untreated dental caries in the permanent dentition was reported as the most prevalent condition in the Global Burden of Diseases Study in 2017 [2]. The disease affects quality of life and may cause pain and discomfort, tooth loss and medical complications [3]. Furthermore, dental caries is an expensive condition to manage, both for the affected individuals and society [4]. Although, studies from Norway indicate a reduction in adult dental caries experience [5, 6], recent epidemiological studies from Northern- Norway found that dental caries is still prevalent among adults [7–9].

WHO CSDH have summarized evidence on health determinants in order to reduce health difference and promote equity [10]. In 2010, WHO CSDH published the conceptual framework for action on the social determinants of health. This action-oriented framework illustrates structural, social and intermediary determinants of health, and how these determinants affect health. Structural determinants, i.e., political, economic, and societal context, in which people live, generate stratification, and determine people’s socioeconomic position (SEP). These structural mechanisms form health opportunities for groups based on their placement within the social hierarchy and produce social inequalities through shaping intermediary determinants, i.e., material and family circumstances, psychosocial, and behavioral factors. The most commonly used SEP indicators related to social health inequalities are educational attainment, income, occupation and social class [11]. These determinants have different causal pathways to health. It has been suggested that educational attainment is a basis and a contributor to processes which influences people’s health [12]. Generally, the highest level of education is established early in the course of life and educational attainment is a relatively unchanging feature comparing for example to income.

Inequality in health often manifests in a gradient [13]. The social gradient in health describes the slope phenomenon when increasing quantities of resources, such as education, correspond with increasing levels of health, in a dose response relationship [14, 15]. Several systematic reviews demonstrated negative association between dental caries and SEP indicators, including educational attainment [16–18]. Some recent studies conducted in Northern- Norway showed association between education level and dental caries experience [7, 8], while one of the study where several social, economic and behavioral determinants was tested simultaneously in a theoretical model failed to demonstrate a direct association [19]. These studies were performed mainly in suburban and rural areas and did not take into the consideration WHO CSDH conceptual framework on social and intermediary determinants of health.

**Fig. 1:**
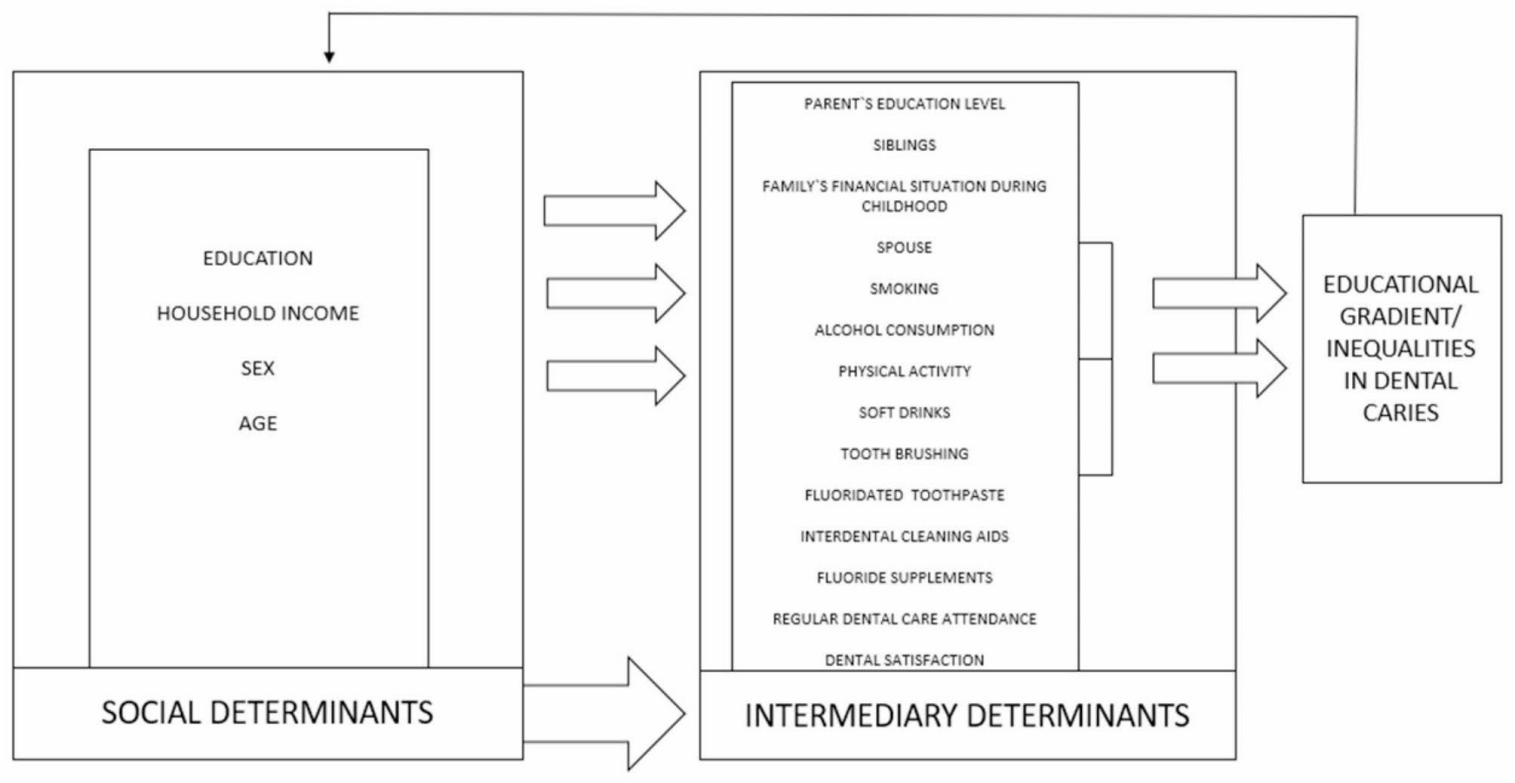
Conceptual framework of the study, based on the WHO CSDH framework on social and intermediary determinants of health

The aim of this study was to assess an association between educational level and dental caries experience in adults in urban Tromsø municipality, Northern-Norway, using WHO CSDH conceptual framework of health determinants (Fig. 1).

## Methods

### Study design and population

This cross-sectional study included data from 3752 participants aged 40–92 years who had data on both dental caries experience and educational level in the seventh survey of the Tromsø Study: Tromsø7. All 32 591 inhabitants of Tromsø municipality aged 40 years or older were invited to participate in the Tromsø7 study [20]. Out of them, 21,083 (65%) attended. The data were collected from March 2015 to November 2016 and included both questionnaires and clinical examinations. A random sample of 3943 (19%) participants were invited to a clinical oral examination [9].

### Variables and measurements

The variables in this study were pre-selected based on the conceptual framework of this study (Fig. 1).

### Outcome variable

The outcome variable, dental caries experience, was expressed as DMFT score [21]. In the assessment of decayed teeth, the classification by Amarante and colleges was employed [22], a five-graded diagnostic scale was used (D_1–2_: decay into enamel; D_3–5_: decay into dentin). The calculation of DMFT score was performed after the clinical oral examination based on bite wing radiographs and intra-oral clinical photographs by seven calibrated dentists [9]. Two calibration tests were conducted, and the dentists had a mean inter- examiner agreement of Cohen’s kappa 0.70 and intra-examiner agreement was 0. 81. For statistical analyses the DMFT score was dichotomized into lower DMFT score (0–19) and higher DMFT score (20–28). The cut-off point was the median DMFT score value in the study sample.

### Exposure variable

Participant’s educational level was self-reported and for the statistical analyses categorized in line with the international standard, ‘primary/partly secondary education’ (up to 10 years of schooling), ‘upper secondary education’ (a minimum of three years), ‘tertiary education, short’ (collage/university less than 4 years) and ‘tertiary education, long’ (collage/university 4 years or more) [23]. The last group was chosen to be the reference category in the binary logistic regression analysis.

### Covariates

#### Social determinants

Social determinants included sex, age and household’s gross taxable income last year. Household income was grouped into four categories: ‘low’ (≤ 450 000 NOK), ‘lower middle’ (451–750 000 NOK), ‘upper middle’ (751- 1 000 000 NOK) and ‘high’ (> 1 000 000 NOK).

#### Intermediary determinants

Family’s circumstances during childhood were depicted by the parents’ education level (categorized in line with the international standard), number of siblings (≤ 2, 3–4 and > 4) and family’s financial situation during childhood (‘difficult’ - very difficult, difficult; and ‘good’ - very good, good). Civil status was recorded as having spouse or not.

Health-related behavior were represented by smoking status (never, yes; now, yes; previously), alcohol consumption (‘never/seldom’ - never, monthly, or more seldom; ‘monthly’ − 2–4 times per month; and ‘weekly’ − 2–3 times per week, 4 or more times per week) and physical activity ‘never/seldom’ - never, less than ones a week; ‘often’ - ones a week, 2–3 times a week; ‘daily - approximately every day).

Oral health-related behaviors used in the model were frequency of soft drinks consumption (‘never/rarely’ - rarely/never and 1–6 times per week; and ‘daily’ − 1 per day, 2–3 per day, ≥ 4 per day), tooth brushing (‘weekly’ - ones a week or more seldom, a couple of times a week; and ‘daily’ - one time a day, two or more daily), use of fluoridated toothpaste (yes/no), interdental cleaning aids (yes/no), use of fluoride supplements (yes/no) and regular dental care attendance (yes/no). Psychosocial intermediary determinants were ‘dental satisfaction’, which was measured on a 5-point Likert scale and categorized into ‘not satisfied’ (1–3) and ‘satisfied’ (4, 5).

### Statistical analysis

Statistical Package for the Social Sciences Version 27.0 software was used for statistical analyses (IBM Corp Armonk, NY, USA). Chi- square test and independent sample t-test were used to analyze differences in distribution of determinants between participants having lower and higher DMFT scores. Univariable binary logistic regression analyses were used to explore association between DMFT score and educational level, as well as other social and intermediary determinants. Multivariable binary logistic regression model was used to explore association between DMFT score and educational level when adjusted for social and intermediary variables which were significant in Table 1. A sensitivity analysis, which included all the variables listed in Table 1, in the multivariable model, was conducted. The level of significance was set at p = 0.05 and odds ratios (OR) are presented with 95% confidence intervals (CI).

**Table 1:** Social and intermediary determinants of dental caries stratified by lower and higher DMFT score in the study sample

Determinants	Lower DMFT 0–19	Higher DMFT 20–28	Missing values n (%)	p-value
**Social determinants**				
**Education level** ^**c**^				**< 0.001** ^**d**^
Primary/partly secondary education	290 (30.8)	652 (69.2)		
Upper secondary education	568 (51.7)	531 (48.3)		
Tertiary education, short	405 (55.3)	328 (44.7)		
Tertiary education, long	705 (72.1)	273 (27.9)		
**Sex** ^**c**^				**0.741** ^**d**^
Female Male	1012 (52.2) 956 (52.7)	927 (47.8) 857 (47.3)		
**Age** ^**a**^	51.8 (8.8)	65.1 (9.6)		**< 0.001** ^**b**^
**Household income** ^**c**^			132 (3.5)	**< 0.001** ^**d**^
Low income	265 (32.8)	544 (67.2)		
Lower middle income	519 (48.4)	554 (67.2)		
Upper middle income	526 (60.7)	341 (39.3)		
High income	626 (71.9)	245 (28.1)		
**Intermediary determinants**				
**Mother’s education** ^**c**^			100 (2.7)	**< 0.001** ^**d**^
Primary/partly secondary education	1233 (45.2)	1494 (54.8)		
Upper secondary education	440 (71.5)	175 (28.5)		
Tertiary education, short	173 (82.0)	38 (18.0)		
Tertiary education, long	87 (87.9)	12 (12.1)		
**Father’s education** ^**c**^			135 (3.6)	**< 0.001** ^**d**^
Primary/partly secondary education	1019 (44.7)	1261 (55.3)		
Upper secondary education	506 (62.1)	309 (37.9)		
Tertiary education, short	234 (75.2)	77 (24.8)		
Tertiary education, long	163 (77.3)	48 (22.7)		
**Siblings** ^**c**^			285 (7.6)	**< 0.001** ^**d**^
≤ 2	1071 (60.6)	696 (39.4)		
≤ 4	549 (50.2)	545 (49.8)		
>4	233 (38.5)	373 (61.5)		
**Childhood financial** ^**c**^ **situation**			88 (2.3)	**< 0.001** ^**d**^
Good	1487 (54.9)	1220 (45.1)		
Diffucult	449 (46.9)	508 (53.1)		
**Spouse** ^**c**^			193 (5.1)	**< 0.001** ^**d**^
Yes No	1501 (54.0) 365 (47.0)	1281 (46.0) 412 (53.9)		
**Smoking** ^**c**^			16 (0.4)	**< 0.001** ^**d**^
Yes, now Yes, previously Never	239 (46.6) 774 (45.4) 950 (62.6)	274 (53.4) 932 (54.6) 567 (37.4)		
**Alcohol consumption** ^**c**^				**< 0.001** ^**d**^
Weekly Monthly Never/seldom	601 (54.6) 818 (55.5) 549 (46.6)	499 (45.4) 656 (45.5) 629 (53.4)		
**Physical activity** ^**c**^				**< 0.001** ^**d**^
Daily Weekly Never/seldom	574 (51.7) 1137 (54.6) 257 (45.8)	536 (48.3) 944 (45.4) 304 (54.2)		
**Soft drinks** ^**c**^			133 (3.5)	**0.942** ^**d**^
Weekly Daily	64 (52.9) 1862 (53.2)	57 (47.1) 1636 (46.8)		
**Tooth brushing** ^**c**^			72 (1.9)	**< 0.001** ^**d**^
Weekly Daily	14 (27.5) 1922 (53.0)	37 (72.5) 1707 (47.0)		
**Fluoridated toothpaste** ^**c**^			127 (3.4)	**< 0.001** ^**d**^
No Yes	149 (31.6) 1776 (56.3)	322 (68.4) 1378 (43.7)		
**Interdental cleaning aids** ^**c**^			130 (3.5)	**0.066** ^**d**^
No Yes	455 (56.0) 1472 (52.4)	357 (43.7) 1338 (47.6)		
**Fluoride tablets** ^**c**^			374 (10.0)	**0.002** ^**d**^
No Yes	1730 (53.4) 91 (66.9)	1512 (46.6) 45 (33.0)		
**Fluoride rinse** ^**c**^			243 (6.5)	**0.520** ^**d**^
No Yes	1565 (53.5) 319 (54.9)	1363 (46.5) 262 (45.0)		
**Dental care attendance** ^**c**^			69 (1.8)	**0.066** ^**d**^
No Yes	165 (48.0) 1775 (53.2)	179 (52.0) 1564 (46.8)		
**Dental satisfaction** ^**c**^			52 (1.4)	**< 0.001** ^**d**^
Satisfied Not satisfied	1224 (58.2) 729 (45.7)	879 (41.8) 868 (54.4)		

Values in the table: ^a^ means (SD) for continuous variables and ^c^ number (%) for categorical variables

^b^ Independent sample t-test

^d^ Pearson’s chi-square test

Subgroups may not be total due to missing values

## Results

### Sample characteristics

This study included 1939 (52%) women. Minimum age for the participants was 40 years and maximum – 92 years, the mean age was 57.11, standard deviation (SD) 11.3. The median DMFT score among all participants was 19, inter quartile range (IQR) 9 and mean 18.03, SD 6.41. Many participants (29%) reported having upper secondary education, 26% of the participants had tertiary education, long and 25% of the participants reported that they had primary/partly secondary education.

In total, 1968 (52.5%) of the participants had lower DMFT score (0–19) and 1784 (47.5%) had higher DMFT score (20–28). The participants having lower versus higher DMFT scores were different regarding their own education level, household income, parents’ education level, number of siblings, smoking habits, alcohol consumption, and physical activity (Table 1). A higher proportion of participants having higher DMFT score were older, had no spouse, had a difficult childhood financial situation, reported to brush teeth weekly, did not use fluoridated toothpaste and fluoride tablets, and was not satisfied with their teeth.

### Education level association with dental caries

According to the univariable binary regression analysis, education level was inversely associated with DMFT score. The participants with lower than secondary education had 5.8-fold increased odds, those with upper secondary education had 2.4-fold increased odds, and those with tertiary education, short had 2.09 -fold increased odds of having higher DMFT score compared to the participants with tertiary education, long (Table 2).

In the multivariable regression analysis, the associations between educational level and having higher DMFT score remained significant (Table 2). The clear educational gradient was not observed between intermediate educational levels. Participants who had lower than secondary education had 2.06 -fold increased odds of having higher DMFT score than those with tertiary education, long (OR: 2.06, 95% CI: 1.50–2.83). Those with upper secondary education had 60% higher odds of having higher DMFT score (OR: 1.60, 95% CI: 1.21–2.11), and those with tertiary education, short had 66% higher odds of having higher DMFT score (OR: 1.66, 95% CI: 1.24–2.22) than those with tertiary education, long, respectively. The results from the sensitivity analysis which included all pre-selected variables were similar to the results from the final multivariable binary logistic regression model (Supplementary Table 1). Supplementary Table 2 shows associations between all covariates and dental caries experience.

**Table 2:** Association between education level and dental caries experience (lower and higher DMFT score)

	Univariable regression		Multivariable regression	
Education level	OR (95% CI)	p-value	OR (95% CI)	p-value
Tertiary education, long	Reference group		Reference group	
Lower than secondary education	5.81 (4.77–7.07)	**< 0.001**	2.06 (1.50–2.83)	**< 0.001**
Upper secondary education	2.41 (2.01–2.90)	**< 0.001**	1.60 (1.21–2.11)	**0.001**
Tertiary education, short	2.09 (1.70–2.56)	**< 0.001**	1.66 (1.24–2.22)	**0.001**

Odds ratio (OR) with 95% Confidence intervals (CI)

The multivariable regression model was adjusted for the following variables: Age, household income, parents’ education level, siblings, childhood financial situation, spouse, smoking, alcohol consumption, physical activity, tooth brushing, fluoride toothpaste, fluoride tablets and dental satisfaction

## Discussion

The current study indicated educational gradient in dental caries experience among adults in Tromsø municipality, Northern- Norway, when using WHO CSDH conceptual framework for health determinants as a basis for analysis. Education level was inversely associated with dental caries experience even after adjustment for social and intermediary health determinants.

Our results are in line with systematic reviews concluding that lower SEP was associated with dental caries experience [16, 17]. One of the systematic reviews also demonstrated that this relation was significantly higher in countries with high compared to low human development index [17]. The present study was performed in Norway, a country with high human development index. Our result are in contrast to a cross- sectional study conducted in the United Kingdom, which did not establish a relationship between educational level and caries experience [24]. Of note, that the aspects of intermediary determinants were not included in the latter study, which might be important in the complex understanding of educational inequalities in dental caries. In addition, a study conducted in Northern- Norway which employed structural equation modelling did not find any direct associations between education and decayed teeth when simultaneously controlling for several social and intermediary factors [19]. However, they used another classification for education than the present study with three categories, middle school, high school or university. The authors argued that the relation between social determinants and dental caries experience might be more complex on an individual level. Indeed, dental caries is a result of a complex interplay between both social and intermediary determinants [3].

Norway is a country known for its equality in educational opportunities, education is free-of-charge and those who would like to pursue higher education are eligible to receive state support. It is challenging to explain educational health inequalities in modern welfare states like Norway. Literature indicates that people with higher education have better living conditions, have more stable employments and less divorces [12]. It is plausible that these mechanisms also promote healthy choices and lifestyle, which is represented by intermediary determinants in the present study. Another possible mechanism may be that people with higher education have more flexible jobs that enable them to adopt health-promoting behaviors like adequate dental hygiene [25]. It is expected that more educated individuals have higher income [26] which allows them to comply with healthy, from an oral perspective, nutrition recommendation. Indeed, the interplay between income and education on health inequalities is not well understood [27]. It has been demonstrated that income inequalities in periodontal health disappeared when the analysis was adjusted for education [24]. The authors argued that education may be a proxy for health behaviors. In our analysis, we followed the WHO CSDH conceptual framework for action on social determinants of health and adjusted for income as one of the social determinants as well as behaviors as intermediary determinant.

Education level may influence the way one chooses to interact with oral health services and use information [24]. Regular dental attendance have been suggested to have a positive impact on oral health [28, 29] and it has been indicated that access to dental care services may weaken the social gradient in oral health through oral health education [30]. In Norway, the government provides free dental services for children and adolescent, but the general adult population pay out of pocket for dental services themselves [31]. It has been demonstrated that there is a social gradient in the use of dental services in Norway [32] and the most important reason for unmet needs for dental care in Norwegian adults has been reported to be economy.

By applying a life course perspective, we might be able to explore how inequalities in health may arise [33]. Oral health inequality in adults might partly be determined by exposure to factors in the beginning of their life which in this study is represented by the variables explaining childhood material circumstances, parents’ education level and numbers of siblings [34, 35]. A population-based study in Northern- Norway demonstrated that adolescent’s own study program was associated with inequalities in dental caries [36]. Consequently, the roots of inequalities in adulthood may lie in inequalities experienced in childhood and adolescence [37].

The recurring theme is the causal relationship between education level and health. The cross-sectional design of the present study, however, does not shed light on this aspect. The WHO CSHD framework suggests that education through intermediate determinants lead to health inequalities. Nevertheless, there are theories suggesting that inferior health may lead to a lower education [12].

The result of the present study supports educational inequalities in dental caries in adults in Northern- Norway and calls researchers and oral health policy makers to give a closer attention to investigations of social oral health inequalities. From a policy perspective, promoting regular dental attendance and reducing barriers to dental care services for lower SEP groups could flatten the slope of the gradient [19]. WHO CSHD highlights the need to focus not exclusively on reducing disease prevalence, but tackling its root, which involves addressing the structural and social mechanisms [10]. Moreover, several of the political, economic, and societal mechanisms lie outside the health sector and can solely be addressed by applying a multisectoral approach. Furthermore, non-communicable health conditions share the same determinants, hence oral health interventions should be integrated with general health promotion and disease prevention strategies [38].

## Conclusion

The results of the current cross-sectional study suggested an educational gradient in dental caries experience in an adult population of Northern- Norway. However, there is a need for more studies to validate our results and investigate mechanisms of education inequalities in oral health.

### Electronic supplementary material

Below is the link to the electronic supplementary material.

Supplementary Material 1

Supplementary Material 2

## Data Availability

The Tromsø study: Tromsø7 datasets used and analyzed during the current study were supplied by “Helsefak ISM Tromsøundersøkelsen” under the agreement and so cannot be made freely available. Requests for access to these data should be made to tromsous@uit.no.

## Abbreviations

CI: Confidence interval
CSDH: The Commission on Social Determinants of Health
DMFT: Decayed, missing, filled, teeth (permanent teeth)
IQR: Inter quartile range
OR: Odds ratio
SD: Standard deviation
SEP: Socioeconomic position
WHO: World Health Organization
