# Supplementary material for: The educational gradient in dental caries experience in Northern- Norway: a cross-sectional study from the seventh survey of the Tromsø study
Source: BMC Oral Health. 2023 Oct 24;23:779. doi: 10.1186/s12903-023-03487-w (PMC10594764; doi:10.1186/s12903-023-03487-w)
Supplement: Supplementary file 1 — Supplementary Material 1 [file 12903_2023_3487_MOESM1_ESM.docx]

Supplementary Table 1- Sensitivity analysis which includes all pre-selected variables listed in Table 1.

|  | **Multivariable regression** |  |
| --- | --- | --- |
| **Education level** | **OR (95% CI)** | **p- value** |
| Tertiary education, long | Reference group | Reference group |
| Lower than secondary education | 2.13 (1.53-2.95) | **<0.001** |
| Upper secondary education | 1.71 (1.29-2.28) | **<0.001** |
| Tertiary education, short | 1.73 (1.29-2.33) | **<0.001** |

^Adjustment variables in the multivariable model with all variables: Sex, age, household income, parent’s education level, siblings, childhood financial situation, spouse, smoking, alcohol consumption, physical activity, soft drinks, tooth brushing, fluoride toothpaste, interdental cleaning aids, fluoride tablets, fluoride rinse, dental care attendance, and dental satisfaction.^
