# Supplementary material for: The educational gradient in dental caries experience in Northern- Norway: a cross-sectional study from the seventh survey of the Tromsø study
Source: BMC Oral Health. 2023 Oct 24;23:779. doi: 10.1186/s12903-023-03487-w (PMC10594764; doi:10.1186/s12903-023-03487-w)
Supplement: Supplementary file 2 — Supplementary Material 2 [file 12903_2023_3487_MOESM2_ESM.docx]

Supplementary Table 2- Associations between social and intermediary determinants and dental caries experience (lower and higher DMFT score) according to multivariable binary logistic regression analysis

| **Multivariable regression** |  |
| --- | --- |
| **Social determinants** |  |
|  | **OR (95% CI)** |
| **Education level** |  |
| Tertiary education, long | Reference group |
| Lower than secondary | 2.06 (1.50-2.83) |
| Upper secondary | 1.60 (1.21-2.11) |
| Tertiary, short | 1.66 (1.24-2.22) |
| **Age** | 1.15 (1.13-1.16) |
| **Homehold income** |  |
| Lower income | Reference group |
| Lower middle income | 0.94 (0.68-0.129) |
| Upper middle income | 1.03 (0.72-1.48) |
| High income | 1.00 (0.80-1.40) |
| **Intermediary determinants** |  |
| **Mother’s education** |  |
| Pimary/partly secondary | Reference group |
| Upper secondary | 0.70 (0.52-0.94) |
| Tertiary education, short | 0.74 (0.45-1.21) |
| Tertiary education, long | 0.49 (0.21-1.11) |
| **Father’s education** |  |
| Pimary/partly secondary | Reference group |
| Upper secondary | 0.91 (0.71-1.16) |
| Tertiary education, short | 0.77 (0.52-1.15) |
| Tertiary education, long | 0.88 (0.52-1.49) |
| **Siblings** |  |
| ≤ 2 | Reference group |
| ≤ 4 | 0.93 (0.75-1.16) |
| >4 | 1.06 (0.80-1.40) |
| **Childhood financial situation** |  |
| Difficult | Reference group |
| Good | 1.12 (0.90-1.41) |
| **Spouse** |  |
| Yes | Reference group |
| No | 1.02 (0.77-1.36) |
| **Smoking** |  |
| Yes, now | Reference group |
| Yes, previously | 0.75 (0.56-1.01) |
| Never | 0.51 (0.38-0.70) |
| **Alcohol comsumption** |  |
| Never/seldom | Reference group |
| Weekly | 0.98 (0.78-1.23) |
| Monthly | 1.13 (0.87-1.47) |
| **Physical activity** |  |
| Never/seldom | Reference group |
| Daily | 0.98 (0.47-1.31) |
| Weekly | 0.98 (0.71-1.35) |
| **Tooth brushing** |  |
| Weekly | Reference group |
| Daily | 0.54 (0.22-1.35) |
| **Fluoridated toothpaste** |  |
| No | Reference group |
| Yes | 0.92 (0.68-1.25) |
| **Fluoride tablets** |  |
| Yes | Reference group |
| No | 1.00 (0.58-1.72) |
| **Dental satisfaction** |  |
| Not satisfied | Reference group |
| Satisfied | 0.59 (0.49-0.72) |

_Odds ratio (OR) with 95% Confidence intervals (CI)._
